# The experience of children using long-term non-invasive ventilation: a qualitative study

**DOI:** 10.3389/frsle.2024.1459349

**Published:** 2024-11-28

**Authors:** Deborah Olmstead, Allison Carroll, Jennifer Klein, Joanna E. MacLean

**Affiliations:** ^1^Respiratory Medicine, Stollery Children's Hospital, Edmonton, AB, Canada; ^2^Women and Children's Health Research Institute, University of Alberta, Edmonton, AB, Canada; ^3^Department of Paediatrics, University of Alberta, Edmonton, AB, Canada; ^4^The Royal Children's Hospital, Melbourne, VIC, Australia; ^5^Glenrose Rehabilitation Hospital, Edmonton, AB, Canada

**Keywords:** adherence, focus group, framework analysis, pre-teens, NIV equipment

## Abstract

**Objectives:**

To identify factors to optimize long-term non-invasive ventilation (LT-NIV) use by exploring the experience of children using LT-NIV and their parents.

**Study design and methods:**

A qualitative framework analysis method was used. Children aged 8–12 years who used LT-NIV for at least 3-months and their parents/guardians were approached to participate. Thematic analysis of data derived from focus group interviews, conducted separately for children and parents, was performed. Findings were coded and grouped into identified themes.

**Results:**

Data analysis identified four themes: (1) “The double-edged sword,” which identified benefits and challenges of LT-NIV use; (2) “Feeling different,” where children and parents described fears, frustrations, and concerns including emotional and social implications, and physical changes; (3) “It's not just about the mask,” highlighted the influence of equipment issues, including the mask interface, headgear, tubing and humidity, and their impact on tolerance and use of LT-NIV; and (4) “Through the eyes of experience—children and parents as experts for change,” which captured ideas for the functional and aesthetic improvement of the equipment including the need for pediatric specific technology.

**Conclusions:**

LT-NIV use has two sides; it helps to improve lives though requires an investment of time and commitment to ensure success. Investing in pediatric-specific equipment needs to be a priority as do alliances between healthcare providers, children who use LT-NIV, and their families. Future technology development and studies of adherence need to consider the experiences of children and their families to reduce the challenges and support optimal use of LT-NIV.

## Introduction

Long-term non-invasive ventilation (LT-NIV), including both continuous and bilevel positive airway pressure (CPAP, BPAP), has become standard of care for children with a wide range of sleep-related breathing disorders and children with chronic respiratory insufficiency or failure (Fauroux et al., 2022; Castro-Codesal et al., 2018a; Windisch et al., 2018). Indications for LT-NIV use have expanded and include children with a broad range of medical complexity (Tan et al., 2020; Pavone et al., 2020; Castro-Codesal et al., 2018b). A scoping review of LT-NIV use in children identified 73 medical conditions for which LT-NIV was used (Castro-Codesal et al., 2018a). While LT-NIV can improve survival, respiratory events and gas exchange, and reduce hospitalization, the need for invasive ventilation, and airway surgery, it also presents challenges for children and their families (Windisch et al., 2018; Hudson et al., 2022; AlBalawi et al., 2022; Bedi et al., 2018a).

Adherence to LT-NIV is challenging for children and adults. The majority of data on adherence stems from studies of CPAP use for obstructive sleep apnea (OSA) and show that the average usage of CPAP for nocturnal users is < $\frac{1}{2}$ the night (Adeleye et al., 2016; Perriol et al., 2019; Machaalani et al., 2016; Patil et al., 2019; Bhattacharjee et al., 2020; Castro-Codesal et al., 2020; Bedi et al., 2018b). Adherence to LT-NIV is a complex issue impacted by a range of factors, including health-related factors, psychosocial circumstances, and the interaction with the technology (Perriol et al., 2019; Pascoe et al., 2019; Katz et al., 2020; Hurvitz et al., 2023). Qualitative studies in adolescents show early adaptation and fewer difficulties around initiation promote continued use while negative experiences, technical and comfort issues, and side effects increase non-adherence risk (Prashad et al., 2013; Ennis et al., 2015; Alebraheem et al., 2018). Whether adolescents' experiences reflect the overall experience of LT-NIV use in children is unclear. The aim of this study is to understand the experience of using LT-NIV from the perspectives of pre-adolescent children and their parents with lived experience. The results will provide insight into improving the ease of use and optimizing adherence in children using LT-NIV.

## Methods

This is a descriptive, qualitative study. Focus group interviews, recognized as a developmentally appropriate and valid approach in children as young as 6 years of age, were utilized for data collection (Kennedy et al., 2001). The study protocol was approved by the Human Research Ethics Board (Pro00050876). All children and their parents/guardians provided informed assent and consent, respectively. The LT-NIV clinic team was composed of respiratory and sleep physicians, a nurse practitioner, and a respiratory therapist. Strategies to establish and support on-going use of LT-NIV include education, mask desensitization, customization of head-gear, and both in-person and virtual follow-up. All children underwent mask selection and fitting in the LT-NIV clinic.

Focus group participants were recruited through a multi-disciplinary tertiary care LT-NIV program. This included all children aged 8–12 years who were established on LT-NIV (i.e., minimum 3-months use outside an acute care setting) and their parents who were invited to participate via a letter sent from the LT-NIV clinic team that included a return-addressed envelope and an email to respond to the study team. Parents who expressed interest were contacted by phone to confirm their participation and the parent's and child's ability to understand and converse in English. After obtaining informed consent and assent, parents completed a health screening questionnaire for their child including information about NIV use with adherence assessed based on the most recent NIV machine download for a period of 2 weeks to 2 months.

Focus groups were conducted in-person and separately for children and parents by a facilitator who had no direct clinical interaction with the participants (AC, JK) using a semi-structured interview guide (see Supplementary material). Focus groups were conducted in a university conference room, were digitally recorded, and transcribed verbatim. Transcripts were anonymized prior to analysis.

Framework analysis, a qualitative method that uses content analysis to capture and organize descriptive data, was chosen for this study. It uses an inductive and iterative approach to data analysis, with findings organized through coding and indexing from within the original dataset (Ward et al., 2013). The interview guide was created by three members of the research team who provide care to children using LT-NIV (DO, AC, and JEM). None of the researchers were parents of children using LT-NIV. During analysis, themes capturing the described experiences of children and their parents were identified along with recurring patterns of common descriptions. Two investigators (AC, DO) independently reviewed the transcripts and completed initial coding of the data. Conceptually related codes were grouped, leading to the emergence of the main themes, which became the analytical framework for data analysis. This framework was refined and applied to each transcript until no new code was identified. Transcripts were repeatedly reviewed by both researchers to ensure consistency within the final coding and themes. Reporting of the results followed the Standards for Reporting Qualitative Research (SRQR; O'Brien et al., 2014).

## Results

Focus groups were completed on 2 separate days with nine child-parent dyads and three parents of children who were unable to participate because of developmental delay. The characteristics of the children using LT-NIV are summarized in Table 1. The most common reason for LT-NIV use was OSA and all but one child had comorbidities. This included three children with asthma, seven with neurodevelopmental disorders (including two with Down syndrome) and one with congenital heart disease. Comparing the characteristics of the children to a description of children using LT-NIV in Alberta, Canada shows that the participants were representative of this population (Castro-Codesal et al., 2018b). Mask fit, mask leak, and skin irritation were rated favoraly by the majority of parents (Table 1). Two parents reported at least occasional skin breakdown with the current mask. Of the 12 parents, 10 were mothers and 2 were fathers. The length of the transcripts was 33 and 45 min for the child focus groups, and 55 and 69 min for the parent focus groups. Data analysis identified 36 codes across four themes.

**Table 1:** Characteristics of the children participating in the focus groups.

***N***	**12**
Age (years)	9.9 (IQR 2.5, range 7–12)
Sex (% female)	42%
**Reason for LT-NIV use**	
Obstructive sleep apnea	8 (68%)
Muscle weakness	1 (8%)
Central sleep apnea	1 (8%)
Central/obstructive sleep apnea	1 (8%)
Chronic lung disease/extreme preterm birth	1 (8%)
**Number of comorbidities**	
None	1 (8%)
One	3 (25%)
Two or more	8 (67%)
Adherence to LT-NIV (hours/night)	9 (IQR 4, range 2–12)
**Mask fit**	
1—poor	1 (8%)
2	0
3	1 (8%)
4	8 (67%)
5—excellent	2 (17%)
**Mask leak**	
1—no leak	4 (33%)
2	4 (33%)
3	3 (25%)
4	1 (8%)
5—large leak	0
**Skin irritation**	
1—no skin irritation	4 (33%)
2	2 (17%)
3	4 (33%)
4	1 (8%)
5—redness lasts all day	1 (8%)

### Theme 1: the “double-edged sword” of using non-invasive ventilation

The children and parents' narratives described the benefits and challenges of NIV use (Table 2). The experience of using LT-NIV was perhaps best elucidated in a parent's quote, “It's a double-edged sword” (P5).

**Table 2:** Representative quotes for theme 1—“the double-edged sword” of using long-term non-invasive ventilation.

	**Child**	**Parent**
Two-sides	“It kinda feels like you're a monster when you wear it, but the good thing is that it helps you” (C4).	“…the blessing and a curse. We hated it at first…and now finally we did find the right mask and he's feeling more rested” (P12)
Benefits	“I get more rest and I do more better at school” (C8).	“It [NIV] has changed our life completely. She was never sleeping well. She would stop breathing… It changed everything. It's been a godsend. She's doing well in school, excited, energy, wanting to do stuff—before it was hard to light a fire under her butt.” (P9)
	“It helps me breathe and it's better than when you go to sleep and you don't have it.” (C2)	
	“If I don't wear my mask I'll get nightmares.” (C3)	“And I'm not kidding you, he went from being the most lethargic, lazy, unenergetic child you ever saw…that first night with BPAP throughout the entire night, he slept 12 h, he woke up the next morning like the energizer bunny…” (P5)
	“At night I wear my mask, I sleep good, not as sleepy.” (C9)	
	“I like it, it feels comfortable, and I just like wearing it.” (C7)	
Challenges	“Just the whole thing in general, just felt weird” (C6)	“To begin with I spent 2 months with her in my arms on the couch in her room to get her to wear the mask.” (P6)
	“It keeps…wakes me up. It scares my dog” (C3)	
	“Well the thing I don't like is I don't like to wear it because I don't like to keep pulling it on and off, like it's really annoying… I like to be able to get out of bed instantly so I can just get a drink of water or something.” (C7)	“I've had a newborn for 12 years…at the beginning I would sleep in his room because I didn't know when his mask would fall on his chin or the hose would come off… I hated it cause I wasn't getting any sleep” (P5).

Children identified that LT-NIV helped them sleep better and feel more rested, although it was not easy to use. Some children focused on the benefits of using their LT-NIV to their sleep as well for their days. Conversely, children expressed their dislike for having to wear LT-NIV, sharing their preference to sleep without it admitting, “If my mom and my dad leaves, I sneak take my mask off.” (C3). They admitted to not always wanting to wear their LT-NIV and wondered whether they would have to use it forever.

Parents shared more about the challenges of first initiating LT-NIV than children. For most, the experience was one of struggle but also determination to be successful. Parents related the impact and challenges, both initially and with on-going use, seeing improvements in their child's sleep, energy, growth, and quality of life. One parent described how her daughter “…is 10 but she's the size of a 5-year-old. So she didn't start to grow til she start to sleep [using NIV].” (P4). Although some children did well from the outset, significant challenges were identified by parents around initiation of LT-NIV. Establishing LT-NIV use could be a long process with one parent describing that “We started with my son in 30 s increments, it took us 2$\frac{1}{2}$ years before he made it to one solid night.” (P5).

Parents identified struggles with their own interrupted sleep and reduced quality of life—not only during the initiation, but also with ongoing requirements for monitoring and readjusting equipment throughout the night. One parent admitted that even after years of use, “I usually check [NIV] every 2 h, I get up” (P6). Reflecting on what it would be like if their child could be successful with LT-NIV, one parent described “…her waking up refreshed on her own, functioning well in the school day and growing and being healthy and happy. I think that's what I'd love to see, and then we could all sleep, which would be miraculous” (P4). Another parent expressed that if their child's LT-NIV therapy were ideal “Life would be glorious. We would have happy children. We would sleep. We would enjoy life” (P5).

### Theme 2: “feeling different” using long-term non-invasive ventilation

Emotional reactions to using LT-NIV were emphasized throughout the focus groups (Table 3). Fears expressed mostly centered around adjusting to LT-NIV therapy itself with descriptions such as being “scared” (C9), “absolutely terrified of [their] mask” (P7) along with the terms “claustrophobic” or “suffocated.”

**Table 3:** Representative quotes for theme 2: “feeling different” using long-term non-invasive ventilation.

	**Child**	**Parent**
Fears	“Kids can get a stroke or they can have a heart attack if they don't keep their mask on ‘til they get up” (C1).	“'Mom, I can't breathe, I can't breathe', and she's crying and scared” (P1)
		“It was brutal…I would cry and I'd say this isn't working”, “he would just scream…mommy I feel like I'm being suffocated” (P12)
	“It kinda sounds like the water's just gonna spill out or something…and then the water's just gonna come up and it's just blowing up my nose…That never happened but I'm just afraid that would happen.” (C7)	“He needs to wear the machine or he's gonna die” (P7)
Physical side effects	“I wake up in the morning and I got blood all over my face. It can happen once a month” (C8)	“He actually has two different masks that we switch once a week cause they found out that his face was growing a little bit different, so we rotate the way the pressures are.” (P6)
	“I don't like to wear it [NIV] because… when I put if off there was just a bunch of dry skin all over my mouth…and every time that happened I have to put on a lip chap, every single time.” (C7)	“I have to keep my son's hair literally in a military cut because [his hair] has got such a memory in it now from all the different headgear. He's actually started to develop a bald spot on his head from the way the fabric of the mask was hitting” (P5)
	“It's just cause all the sweat and the moisture that comes up and it just builds up right there and you get rashes on your face and stuff” (C4).	
Social impact	“If you put a lock on your mask it'll be stuck on you forever…that's a bad thing. Then you'd have to wear it to school. And then your friends would laugh at you” (C3)	“Everybody's like “what's wrong with his head?” … so you always try and keep it [hair] short because then he doesn't look so silly ‘cause he's like ‘kids are laughing at me.” (P3)
	“Your friends probably will laugh at you [if they know about NIV]” (C5)	“With sleepovers, he was embarrassed because he didn't feel like he's a normal kid.” (P12)
	(Question: Do you bring your CPAP with you?) “Yeah except like sleepovers with friend.” (C8)	“As he goes into those horrible teenage years, how's that gonna affect his growth, his image with other kids, all those types of things?” (P5)

Frustrations primarily focused on the physical side effects experienced, including hair changes, nosebleeds, facial irritation, and rashes. Parental accounts mostly reflect concerns regarding the long-term consequences of physical changes. This included facial marking and changes in facial shape with one mother sharing “He's actually started to develop a bald spot on his head from the way the fabric of the mask is hitting. And seeing my son develop a bald spot nearly broke my heart” (P5). Several parents expressed frustration related to healthcare providers not understanding the impact of LT-NIV therapy and giving inconsistent medical advice. One mother shared, “I think sometimes the doctors don't understand what it's like to be a mother or father of a child with these issues because they don't have a child themselves to relate to, or they have never strapped one even on their own face to understand what the child's going through” (P5).

Children and parents expressed further concerns about the potential social impact of using LT-NIV. Being seen as different and admitting to hiding their LT-NIV use from friends was shared by several children through statements such as, “I haven't told them [friends] cause I'm new at school” (C1). Similar concerns expressed by parents include noticing “She hides her stuff when friends come over. It makes her feel different” (P8). Another parent noted “He gets some anxiety about people seeing that…I think that because people say what's that from, why do you have that rash on your face…they're noticing now whereas before they didn't really notice…I think that for him that part of it kinda bothers him” (P11). Parents also anticipated that social concerns might increase in the teenage years.

### Theme 3: it's not just about the mask—the challenges of non-invasive ventilation equipment

Disclosure on the challenges with LT-NIV use for children often centered around masks, machines, and associated equipment (Table 4). Although mask issues were at the forefront, headgear, tubing, and humidity issues were also described by both children and parents.

**Table 4:** Representative quotes for theme 3: it's not just about the mask—the challenges of non-invasive ventilation equipment.

	**Child**	**Parent**
Interface	“I never wanna use the mask” (C5)	“We've tried over 9 years probably 10 different masks, where I've actually just been literally pulling my hair out by the seams because he's just not adjusting to it… I wish I'd had it [new pediatric mask] 9 years ago, I'd be in a different place in time I think.” (P5)
	“I don't like using it [the mask]” (C4)	
	“If I move my mask it keeps on leaking. I just have to stay still.” (C3)	“The new mask he's on now, he doesn't wake us up in the middle of the night if he has to go to the bathroom, he just unclips it, goes to the bathroom, clips it back on again, sets it himself” (P3).
	“I don't know why, but sometimes my mask is sealed perfectly but then air from the little holes at the front just blows up to my eyes and then that's when I can't sleep.” (C6)	“We went through a series of masks; two different machines. Finally found this mask—he wears it all night long and doesn't complain.” (P12)
	“Even my mask was trying to take off last night.” (C3)	“We had 7 different masks to try—cutting headgear apart—changing it up—…. I modify it myself.” (P10)
	“The…about my mask is it bothers me as I'm sleeping.” (C9)	
Headgear, hose, and humidity	“It [tubing] just gets in my way when I'm sleeping sometimes…It bugs me” (C6)	“If I don't cinch it [the mask] down really good, so it seems to already be pushing in, it leaks. So if I loosen it like I've been told it should be looser, so it's just sitting on the face, as soon as she rolls over, it pops out.” (P4)
	“When you roll over some parts of it [headgear] digs into your head and then it hurts in the morning. I tell my mom, then she moves it and then sometimes it works and sometimes it doesn't” (C2).	“And I think, for him, he rolls around too much and the hose is too short. On one mask, it has a little extension on it which seems to work, but when we put it on the other mask it's shorter, and I think it pulls and he feels that air and he just pulls it off.” (P6)
	“Tubing makes the noise, that's what I hate, every time I turn around it's like oh my goodness, I hate this” (C8).	“But he was saying even this morning that with the nasal… he does get irritation, that it really bothers him that it builds up moisture in there even with changing the humidity up and changing it down.” (P2)
	“I almost broke the machine ‘cause when I was trying to sleep, the hose went too long, pushed it down, went on the ground and then it [the machine] was smashed.” (C1)	

Several children shared not liking their masks. A common thread was the number of masks that a child had to try before achieving a suitable fit. Parents did recognize that getting the right equipment for their child was important, but the journey to get there could be long. Mask leaks were a common discussion point. The arrival of newer pediatric masks was described as having a positive impact on tolerance for most children. Headgear also generated discussion among children and parents and was often linked to physical changes caused by the mask fit.

NIV tubing and humidity issues were commonly described by children and parents as having a significant effect on tolerance. One child explained, “Look I hate the hose because every time I try to fall asleep the hose pops off and I don't even notice. And it goes way, way, I'm like what? I'm like Dad. I scream Dad” (C3). Side effects attributed to humidity levels were described by both children and parents. Excess humidity contributed to facial rashes and irritation, with nosebleeds occurring in several children and attributed to low humidity.

### Theme 4: through the eyes of experience—children and parents as experts for change

**Figure 1:**
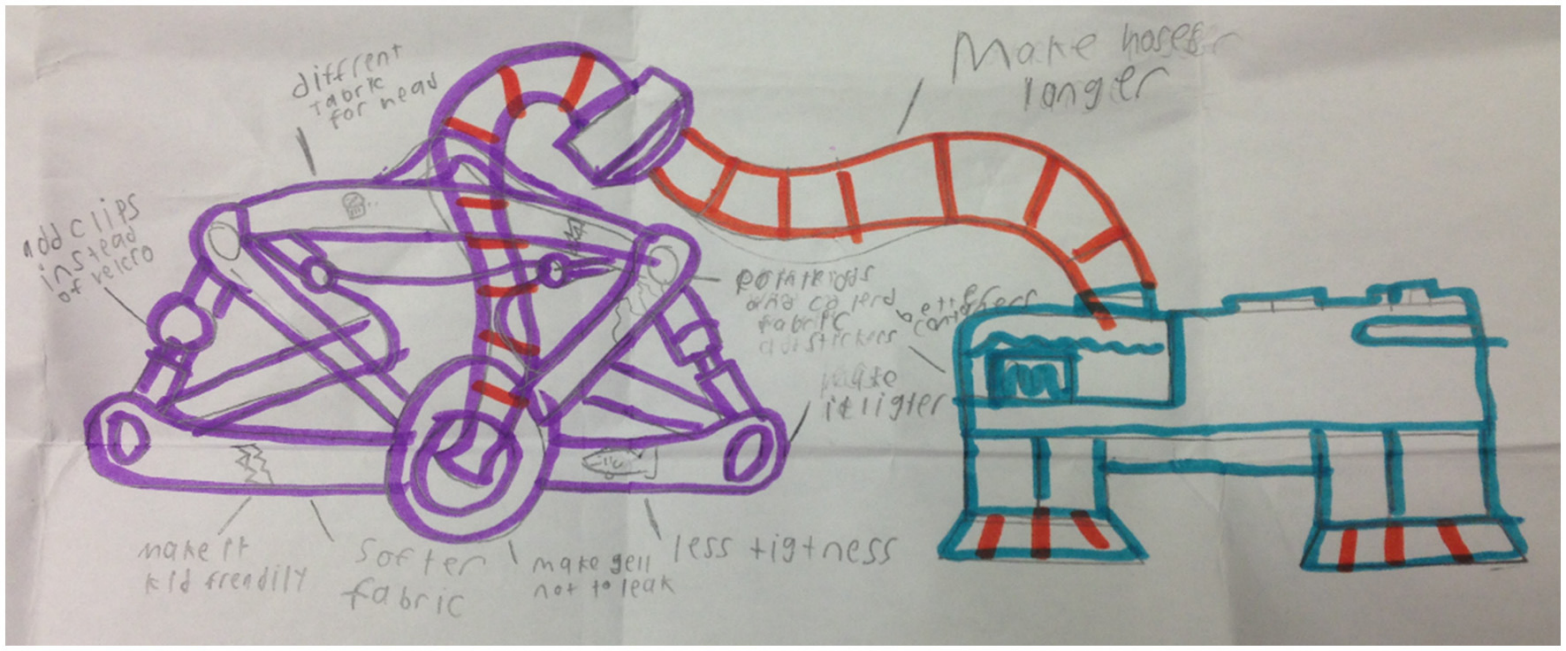
“My ideal BPAP equipment” drawn by 11-year-old study participant (consent obtained to reproduce).

Children and parents had many suggestions for improving LT-NIV technology (Table 5). Children had ideas for child-friendly designs that focused on the aesthetics of NIV equipment. Other discussion focused on the entertainment potential with children suggesting incorporating radios, speakers, and video players, while others recommended choosing colors, stickers and cartoon characters. Other suggestions focused on improving the comfort or side effects of the mask. These included making the headgear and mask softer, preventing skin irritation from the mask and humidity, and modifying the tubing so it did not come apart. A drawing depicting one child's vision of the perfect NIV equipment reflects thoughtful and exacting detail based on his experience using NIV (Figure 1).

**Table 5:** Representative quotes for theme 4: through the eyes of experience—children and parents as experts for change.

**Child**	**Parent**
“I'd probably have side attachments to the mask that would have mini speakers.” (C4)	“Look at it through the eyes of a child, not through the eyes of an adult.” (P5)
“The idea is kids don't wanna wear the mask unless it has ideas like a printed fabric, so have printed fabric with maybe cars on it or flames on it, or something for kids that don't wanna wear it, then they can have a little excitement for the ritual… I would strap Lego guys, on the top strap or maybe stick like Minecraft pictures or something…”” (C3)	“So today, I think everybody getting together and saying yes, we need custom masks made for our kids, I think it's the perfect solution.” (P5); “I totally agree because the off the shelf masks for kids, they just, there's not enough variety or finessing with the masks.” (P4)
“Have music attachments so then you could listen to music and calm yourself down” (C5).	“Our children pretty much need custom ‘cause everyone is to different, you can't put one mask—they need their own custom made headgear to go along with the mask that works for them. To make it easier for us parents.” (P12)
“It would definitely be pink and it would fit properly, it wouldn't fit just like a normal mask, it would fit properly. And different colors and different kinds of stickers” (C2)	“I would like to see them make the masks so that kids are intrigued by them, and that they want to wear them….if the headgear was [NHL hockey team], or it has skulls on it, or I don't know, Monsters University, something custom that the kids would be like OK, I'm gonna go to bed a princess tonight, I'm going to bed a superhero, that maybe they would actually wanna wear it.” (P5)
“Probably so I could just crawl into bed and then you don't have to turn it on, it'll turn on automatically.” (C6)	“Wouldn't it be neat if it was standardized [machine and masks]. So that we could all take a look at the different machines available, the different tubings available, the different mask and headgears available.” (P10)
“I just wish that you could just plug it in, just let it charge, and then like when it's done you could unplug it and then like it just goes by itself. Yeah because like I need it because every time we have to move my little shelf thing to just plug it in.” (C7)	

In contrast, parents focused more on the need for equipment designed to improve their child's acceptance and tolerance of LT-NIV. Parents' suggestions focused on proper pediatric-sized masks and minimizing the side effects of LT-NIV. They generally felt that the current LT-NIV equipment was insufficient to meet the needs of their children. The need for comfortable, well-fitting pediatric-specific equipment with child friendly designs was important to parents.

## Discussion

Despite the challenges and struggles captured within the narratives, both children and their parents revealed underlying perseverance and resiliency. LT-NIV initiation and adherence requires commitment and time from children and their parents to overcome significant emotional, physical, and social challenges associated with LT-NIV use. Even with discussion of significant negative experiences with LT-NIV and equipment challenges, participants reported benefits of LT-NIV.

The “double-edged sword” identified by both children and parents in this study echoes findings from other studies of the experience of children using home mechanical ventilation (HMV). For children using HMV, including NIV and invasive mechanical ventilation (IMV), society relies on family caregivers to provide skilled, vigilant, sometimes around the clock care with families “daily living with distress and enrichment” (Keilty and Daniels, 2018; Carnevale et al., 2006). A meta-synthesis of 12 studies described the experience of life with a child dependent on a ventilator at home (Lindahl and Lindblad, 2011). For the children, this included the risk of being excluded from society and everyday relationships, living with a machine, and thoughts about health and being alive. Parents identified an awareness of reality and the values of life while also recognizing a demanding parenthood that included a lot of learning. In a study of people with neuromuscular disease using NIV, participants identified that NIV had extended their life though their life was different from before NIV with some restoration of some aspects they had lost (Perry et al., 2022). As the sum of the benefits and challenges of LT-NIV use may not always balance, understanding where this balance sits for an individual child and their family is important to consider (Alebraheem et al., 2018). Parents in the current study could see a better life for themselves and their children if only LT-NIV use could be optimized. Documenting the desired benefits as well as a realistic assessment of challenges prior to starting LT-NIV may help to set expectations for the child, family, and healthcare practitioners.

Even in this younger pre-teen age group, feeling different because of LT-NIV was an obstacle. While this included fear and frustration related to NIV use, it also included concerns regarding social relationships and physical appearance. Seeking to be “normal” was a theme in a study of families with a child using HMV where themes did not differ between families whose children used NIV or IMV (Carnevale et al., 2006). In this same study, families identified being offended by the reaction of others to their child using HMV leaving families feeling like strangers in their own communities. Social pressure may help and hinder adherence. In a study of adolescents, those with high CPAP use reported wanting to alleviate worry for their caregivers while those with low or no use reported wanting to be like their peers, with caregivers noting the adolescent's desire to conform to peer norms (Prashad et al., 2013). Friendships are important to children using HMV and wanting to be like their peers is appropriate; keeping their HMV use hidden from others is a way to ensure children using HMV are seen as similar to their peers (Carnevale et al., 2006; Earle et al., 2006). Both the current study results and others highlight feelings of shame, embarrassment, and not being cool related to peers finding out about their HMV use (Prashad et al., 2013; Ennis et al., 2015; Earle et al., 2006). The present study also adds concerns about physical appearance impacted by LT-NIV use, including changes to facial shape, facial marking, and hair loss raised by both children and their parents. While concerns about feeling different may increase in adolescence, addressing these concerns and being vigilant about recognizing physical changes related to LT-NIV use may help to address important barriers to LT-NIV.

Anyone working with LT-NIV users will identify NIV interface or mask concerns as a barrier to consistent LT-NIV use; what may be surprising is that the interface is one of many equipment issues. A well-fitting mask, lower air leak, and getting the right mask early on in LT-NIV initiation are all factors associated with higher adherence (Bhattacharjee et al., 2020; MacDonagh et al., 2021; Bachour et al., 2016) whereas an uncomfortable mask is a common barrier to adherence (Pascoe et al., 2019). Masks for children are often smaller versions of adult masks despite age being an important determinants of facial proportions (Farnell et al., 2021). A recent American Thoracic Society Workshop Report focused on promoting individualize mask selection to improve comfort, adherence and efficacy of CPAP (Genta et al., 2020). Studies of different flow-generators, or device modes (e.g., CPAP vs. AutoPAP), and what have been deemed comfort settings (e.g., humidification, heated tubing, ramp, and expiratory pressure relief) have failed to show benefit for adherence in adults (Killick and Marshall, 2021; Johnson, 2022; Zhuang et al., 2010) although they may improve comfort and adherence for individual children and adults. The current study adds headgear and tubing concerns to the list of potential LT-NIV equipment issues that may affect usage. Suggestions for equipment improvements came from both children and parents, focusing on making the equipment child-friendly and addressing technical issues. With customized masks not yet a practical option (Wu et al., 2018; Duong et al., 2021; Green, 2020), integrating LT-NIV users, including children and their caregivers, into the development and design process of NIV equipment may be the best option for improving user experience with this technology.

The limitations of the study may impact interpretation and translation of the findings. Children and parents who responded to the study invitation may not be representative of all children using LT-NIV and their parents. Children who were unsuccessful at initiating LT-NIV were excluded and those who chose to participate skew toward those with high adherence and good mask fit. Despite this, the results highlight that LT-NIV use presents considerable challenges, even for families who are successful in using this technology. Although small sample size is inherent in most qualitative methods, participants selected purposefully for their contributions in a specific setting are generalizable to any setting in which the problem or situation is a shared experience, such as LT-NIV (Morse, 2016). The experience of the research team members with LT-NIV may have led to bias in the focus group guide and construction of themes. The guide, however, focused on adherence and the interface, neither of which came out as major themes.

## Conclusion

The voices of children and their parents provide a meaningful and necessary perspective on the experience of using LT-NIV. Their accounts detail how LT-NIV has improved their lives by helping children breathe during sleep while highlighting struggles encountered by children and parents including the significant time and commitment invested to ensure successful LT-NIV use. The development of anticipatory guidance and targeted programming for children initiating LT-NIV needs to be informed by the experience of children using LT-NIV. This means including children using LT-NIV and their caregivers in designing the solutions to the challenges of using this technology rather than asking them to test possible solutions after they have been developed. Investing in pediatric-specific equipment needs to be prioritized and children using LT-NIV and their caregivers are the most important stakeholders to be included in the development of guidance to inform policy development for funding and healthcare groups. Ultimately, alliances between health care teams, children and their families will be critical to improve the successful use and health outcomes for children using LT-NIV and their families.

## Data Availability

The raw data supporting the conclusions of this article will be made available by the authors, without undue reservation.
