# Supplementary material for: The experience of children using long-term non-invasive ventilation: a qualitative study
Source: Front Sleep. 2024 Nov 28;3:1459349. doi: 10.3389/frsle.2024.1459349 (PMC12713816; doi:10.3389/frsle.2024.1459349)
Supplement: Supplementary file 1 [file Table_1.DOCX]

On-line supplement

**The experience of children using long-term non-invasive ventilation: a qualitative study**

Deborah Olmstead, Allison Carroll, Jennifer Klein, Joanna E MacLean

**Focus Group Guide**

**Compliance and Mask Issues in Pediatric Non-Invasive Positive Pressure Ventilation: A Focus Group**

**Goals:**

Increase understanding of factors influencing compliance in NIV use in children

Identify specific mask factors that may influence the comfort and compliance of NIPPV by children

| **Activity** | **Time Allotted – 90 minutes** |
| --- | --- |
| Welcome (Name tags, Consent, Demographic Survey) | 15 minutes |
| Introductions, Ground Rules and Icebreaker | 15 minutes |
| Guided Discussion | 45 minutes |
| Wrap-up and thank you | 15 minutes |

**Timeline:**

**Guided Discussion:**

***Children’s Group***

Compliance

What do you like about using your BiPAP or CPAP?

What do you not like about using your BiPAP or CPAP?

What are the biggest reasons why you don’t use your BiPAP or CPAP sometimes?

How do you make it easier?

What makes it difficult to use?

Mask Issues

Can you describe what your mask feels like.

What do you like about your mask?

What makes your mask comfortable/feel good? What makes you want to wear it?

What do you not like about your mask?

What makes your mask uncomfortable/feel bad/feel like you don’t want to wear it?

How would you make the mask better? If you could make changes to your mask that would make you like it better, what would they be?

If you were to design the best mask ever, what would it look like? How would it feel? What special features would it need?

***Parent’s Group***

Compliance

What has been your experience with your child wearing CPAP/BiPAP? If you were going to tell another parent what it was like, how would you describe having your child on NIPPV?

What have you found to be the most challenging aspect to using NIPPV in your child? i.e. the equipment (mask, machine), the schedule, compliance?

What would you describe as the most positive aspects of your child’s use of CPAP or BiPAP?

What do you not like about your child using BiPAP or CPAP?

What difficulties do you encounter? What would you say are the greatest challenges with your child using NIPPV?

When your child does not wear their NIPPV, what are the most common reasons for this?

What strategies have you tried that made it easier for your child to wear their BiPAP or CPAP on a regular basis?

Mask Issues

What has been your experience with your child’s mask?

What do you like about the mask?

What do you not like about the mask?

What have been the challenges encountered with your child’s mask?

How would you change the mask to make it better?

How would you change the whole set-up (ventilator, tubing etc.) to make it better?

If you were to design the best mask ever, what would it look like? What features would it need?

If your child no longer needed to use NIPPV for sleep, how would your life be different?
